# Medicinal Plants Used by Traditional Healers in Algeria: A Multiregional Ethnobotanical Study

**DOI:** 10.3389/fphar.2021.760492

**Published:** 2021-11-29

**Authors:** Khadidja Belhouala, Bachir Benarba

**Affiliations:** Laboratory Research on Biological Systems and Geomatics, Faculty of Nature and Life Sciences, University of Mascara, Mascara, Algeria

**Keywords:** Algeria, medicinal plants (herbal drugs), traditional healers, phytotherapy, ethnobotany

## Abstract

Traditional medicine is the cornerstone that boosts scientific research to explore new therapeutic approaches. The study aimed to assess the traditional knowledge and use of medicinal plants to treat various ailments by Algerian traditional healers. Forty traditional healers were face-to-face interviewed in three different Algerian areas (West, Kabylia, and Sahara). The data collected were analyzed using quantitative indices such as fidelity level (FL) and informant consensus factor (F_IC_). A total of 167 species belonging to 70 families were recorded. Lamiaceae (13%), Asteraceae (13%), Apiaceae (7%), and Rosaceae and Fabaceae (5% each) were the most cited families. The survey revealed that leaves were the most used parts of the plants (29%). Furthermore, decoction (35%), raw (24%), and infusion (19%) were the common modes for the remedies’ preparation. Here, 15% of the total species were newly reported as medicinal plants. Besides, it was reported for the first time a total of 47 new therapeutic uses for 20 known plant species. Of 17 ailments categories, cancer was presented by 44 species, showing the highest F_IC_ of 0.46. *Marrubium vulgare* L.*, Artemisia herba-alba* Asso.*, Zingiber officinale* Roscoe., and *Juniperus phoenicea* L. recorded the maximum fidelity value of 100%. Therefore, our study reveals strong ethnomedicinal knowledge shared by local populations living in the three regions studied. The medicinal species with a high FL could be promising candidates for identifying new bioactive molecules.

## Introduction

Medicinal plants are still considered important and promising sources of drugs to treat various diseases. Their therapeutic uses, vernacular names, modes of preparation, and routes of administration were orally transmitted to constitute a local ancestral knowledge characterizing each population or ethnic group living in a specified area. Actually, from the identification of morphine in opium in the 19th century, drug discovery is based on ethnobotanical investigations and local ethnomedicinal knowledge (Ojah, 2020). Moreover, almost 35% of drugs and about 80% of anticancer drugs used in clinical practice are plants- or natural products-derived (Calixto, 2019).

Algeria is the largest country in the Mediterranean basin, Africa, and the Arab region with a total area of almost 2.4 million km^2^ and 1,600 of coastline. In addition to a diversified climate, Algeria is characterized by a rich flora consisting of 4,000 taxa, 917 genera, and 131 families. Moreover, owing to its ancient history as one of the first cradles of *Homo sapiens* and civilization in the world, Algeria possesses an important and rich cultural diversity. Although several studies have been undertaken to document the local knowledge regarding the use of medicinal plants to treat different diseases (Benarba, 2015; Benarba et al., 2016; Chelghoum et al., 2021; Mechaala et al., 2021), the Algerian ancestral ethnomedicinal knowledge deserves more ethnobotanical investigations. On the other hand, almost all of these ethnobotanical studies covered one region and therefore the same culture and traditions. The present study was carried out in three important regions of Algeria: North-West, Kabylia (Center), and Sahara (South) to 1) record the medicinal species used for medicinal purposes and the local therapeutic practices of traditional healers and 2) document the species newly reported as medicinal plants and new uses.

## Material and Methods

### Description of the Study Area

**FIGURE 1:**
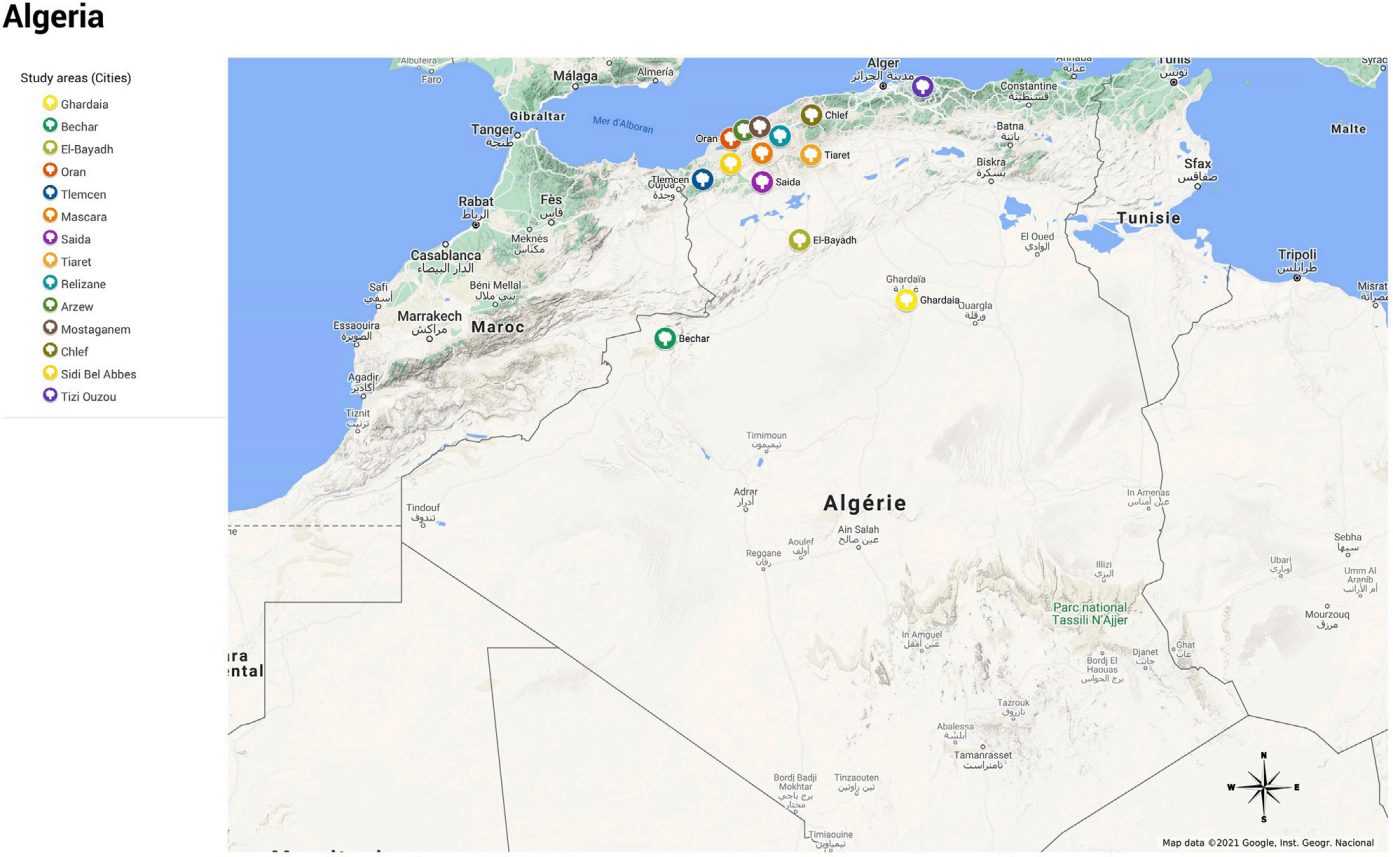
Study areas.

The multiregional study was carried out in three regions in Algeria: North-West, Kabylia (Center), and Sahara (South) (Figure 1). The ethnobotanical investigations in the North-West were performed in five departments: Mascara (area = 5,139 Km^2^), Oran (area = 2,114 Km^2^), Mostaganem (area = 2,269 Km^2^), Sid Bel Abbas (area = 9,150 Km^2^), and Tiaret (area = 20,673 Km^2^) and their surrounding villages located from the Mediterranean Sea to the Moroccan borders. Although no data is available regarding the flora of each department, that of the region of Oran showed the presence of 92 taxa; out of them, 72 remain endemic (Miara et al., 2018). The ethnobotanical study carried out in Center Algeria covered one city named Tizi Ouzou and its surrounding villages covering an area of 3,568 Km^2^, located 100 km east of the capital (Algiers) and 30 km south of the Mediterranean Sea. Owing to its favorable climate, this region is characterized by an important vegetal diversity, including 659 species, 95 subspecies, 2 varieties, and 1 forma from 381 genera and 88 botanical families (Meddour and Sahar, 2021). The south areas included in the present study covered three of the main cities of the Algerian large desert: Ghardaïa (area = 32,256 Km^2^), Bechar (area = 161,400 Km^2^), and El Bayad (area = 71,686 Km^2^), characterized by important cultural, ecological, climatic, and botanical diversity (Taïbi et al., 2020; Taïbi et al., 2021). This desert wide region is characterized by sparse vegetation, grasses appearing during a short period of the year, and rare trees. According to its adaptation mode to the drought, Saharan flora can be divided into ephemeral plants, called “achebs” with a short vegetative cycle of one to four months, and perennial plants with morphological and anatomical adaptations based on an enhanced absorbent system and reduced evaporating surface. The local flora comprises 130 species belonging to 40 families (Chehma and Djebbar, 2008).

### Data Collection

The ethnobotanical investigations were carried out from December 2019 to June 2020. During this period, we visited 13 cities and 19 villages in the study areas, searching for traditional healers. The data had been gathered from 40 informants; 87.5% of them were professionals, acquiring the therapeutic knowledge by the transition from generation to generation, and 12.5% were herbalists. The traditional healers were interviewed by a face-to-face interview in their homes or workplaces to fill out a questionnaire and collect the data. The responses included the demographic characteristics of healers (Table 1) and other information related to the uses of medicinal plants, such as the vernacular name, ailments treated, parts used, preparation, and administration modes. The species were given in their local names in Arabic or Amazigh.

**TABLE 1:** Demographic characteristics of the traditional healers.

Gender	n	100%
F	23	57.5%
M	17	42.5%
Areas		
West	26	65.0%
Kabylia	6	15.0%
Sahara (desert)	8	20.0%
Age		
34–49	4	10.0%
50–65	11	27.5%
66–81	15	37.5%
82–98	10	25.0%
Education		
Illiterate	27	67.5%
Literate	13	32.5%
Inherited	28	70.0%
Acquired	7	17.5%
Unknown	5	12.5%

### Botanical Identification

The medicinal species mentioned by the traditional healers were collected, coded, and dried. Voucher specimens were deposited at the Herbarium of the Laboratory of Research on Biological Systems and Geomatics (LRSBG), University of Mascara, Algeria.

The taxonomic identification was performed by Professor Bachir Benarba using the standard literature (Baba Aissa, 1999; Kunkele and Lohmeyer, 2007; Trabut, 2015).

### Ailment Categories

Table 2 shows more than 100 diseases recorded from the ethnobotanical investigations. All the ailments were classified into 17 categories based on the vital system/organ affected or type of damage.

**TABLE 2:** Ailments categories.

Category	Ailments/disorders	Abbreviation
Kidney diseases	Kidney failure, kidney problems, and urolithiasis	KD
Gastrointestinal system diseases	Irritable bowel syndrome (IBS), ulcers, heartburn, hemorrhoids, stomach ache, diarrhea, constipation, colitis, flatulence, gastrointestinal diseases, gallstones, liver diseases, and jaundice/icterus	GISD
Skin diseases	Limb swelling, itchy skin, tinea capitis, scalp ringworm, heel fissures, skin diseases and ulcer, urticaria, lichen, chalazion, albinism, dermatitis or eczema, boils, head ulcers, skin ulcers, leprosy, festering wounds, and burns	SD
Cancer	Cancer, blood cancer, gum tumors, tumors, skin pimples, uterine cysts/tumors, breast cysts, breast tumors lung tumors, liver cancer, breast cancer, legs cancer, skin cancer, early stage cancer, and stomach cancer	Can
Endocrine system diseases	Goiter and diabetes	ESD
Respiratory tract diseases	Sinusitis, bronchitis, nasal-lung inflammation, pneumonia, lung filtering/smoker, chest and lung diseases, cough, pulmonary-breathing problem, asthma, allergy, cold, and chest pain	RTD
Skeletomuscular system disorder	Osteoarthritis, bones pain, acute arthritis, gout, back pain, arthritis, arthrosis, fracture, osteoporosis, and moving difficulty	SMSD
Cardiovascular system diseases	Cardiovascular diseases, hypertension, clogged arteries, and hypercholesterolemia	CVSD
General health	Earache and deafness, hoarseness, sore throat, fever, mouth ulcer, halitosis, gingivitis, anxiety disorders, and hypochondria, tonsillitis, and incurable diseases	GH
Haircare	Baldness, alopecia areata, and hair loss	HC
Nervous system	Migraine, headache, dizziness, head problems, psychosis, insomnia, epilepsy, and sciatica	NS
Sexual-reproductive problems	Uterine problems, uterine microbe, infections, infertility, breast milk outage, and prostatitis	SRP
Infectious diseases	Laryngitis	ID
Poisoning	Scorpion sting and poisoning	P
Hematological system diseases	Anemia, spleen diseases, and blood purification	HSD
Urology system diseases	Bladder disease, urinary tract infection/inflammation, and cystolithiasis	USD

### Data Analysis

Ethnobotanical indices, fidelity level (FL) and informant consensus factor (F_IC_), were calculated to analyze the data obtained. Consensus indicators FL and F_IC_ were used to quantify the relevance and importance of a species for a given ailment category and the agreement of its use among healers, respectively (Hoffman and Gallaher, 2007; Khan et al., 2014). FL and F_IC_ were calculated using the following formulas (Morvin Yabesh et al., 2014):

**Fidelity level**: FL (%) = (Np/N)*100

*Np* is the number of use reports for a given species reported for a particular ailment category, and *N* is the total number of use reports cited for any given species.

**Informant Consensus Factor**: F_IC_ = (Nur-Nt)/(Nur-1)

Nur is the number of use citations in each category, and Nt is the number of species reported in each category.

## Results

### Botanical Diversity, Parts Used, Modes of Preparation, and Administration

**FIGURE 2:**
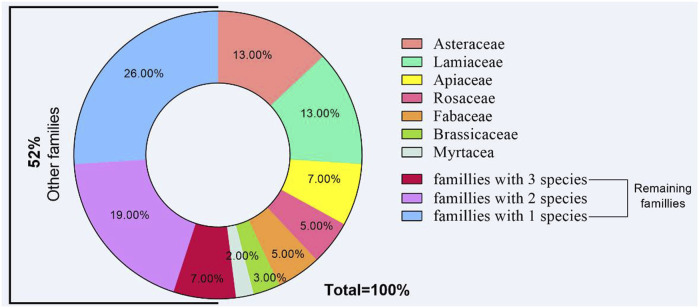
Distribution of reported species among the botanical families.

**FIGURE 3:**
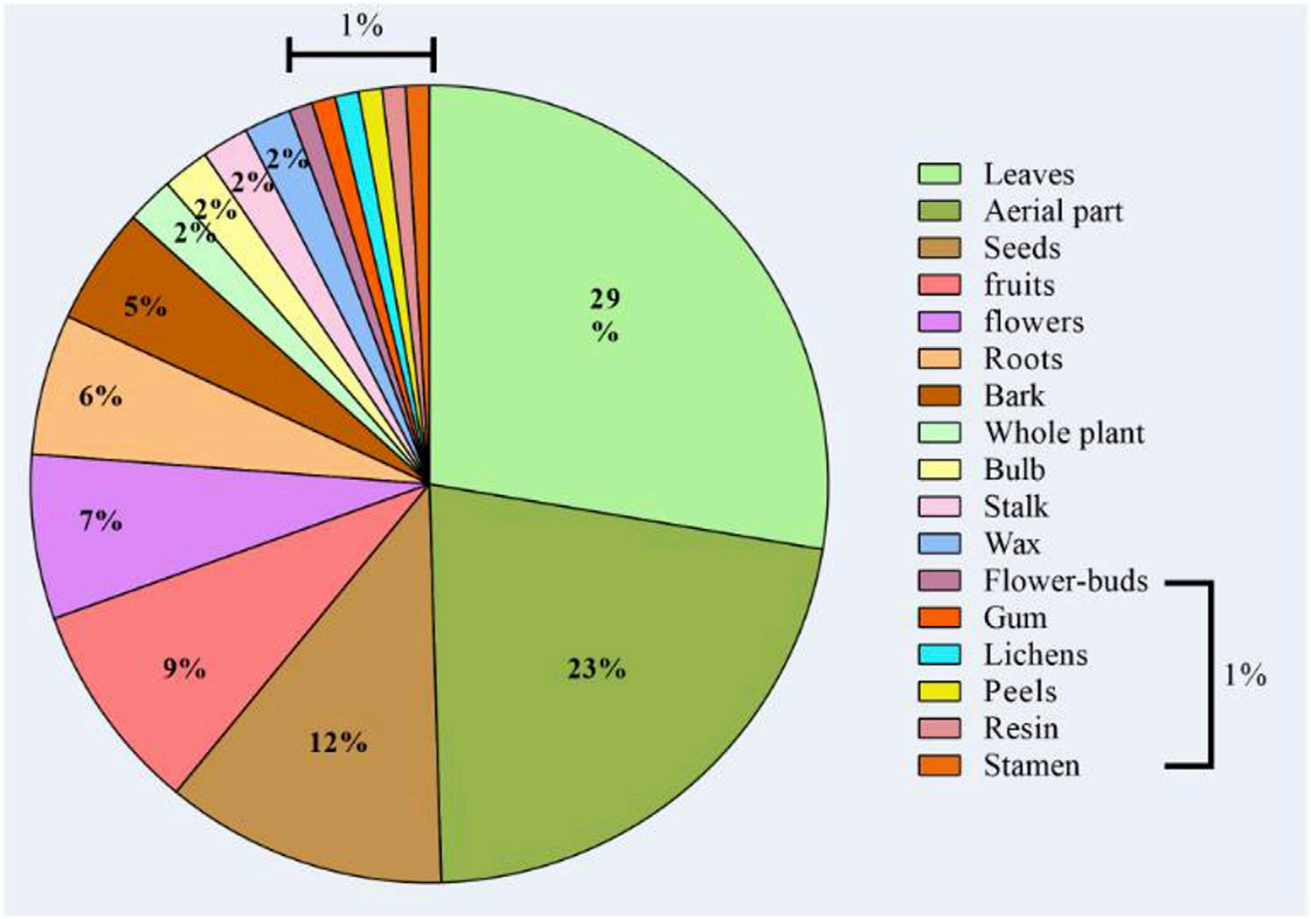
Plants parts used by traditional healers.

This study revealed 167 species of medicinal species used for therapeutic purposes, belonging to 70 families. Lamiaceae (13%), Asteraceae (13%), Apiaceae (7%), Rosaceae (5%), and Fabaceae (5%) were the most cited families, while the 66 remaining families (57%) had between 1 and 5 species in each (Figure 2). As shown in Figure 3, the plant parts most frequently used were leaves (29%), followed by aerial part (23%), seeds (12%), fruits (9%), and flowers (7%). Some used parts were lower than those, such as roots (6%), bark (5%), and whole plant, bulb, wax, and stalk (2% each). Besides, peels, flower buds, stamen, and gum were slightly used (1%).

**FIGURE 4:**
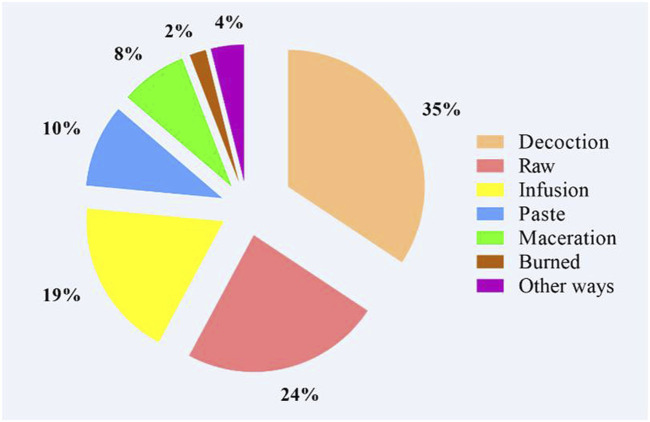
Modes of preparation used by traditional healers.

**FIGURE 5:**
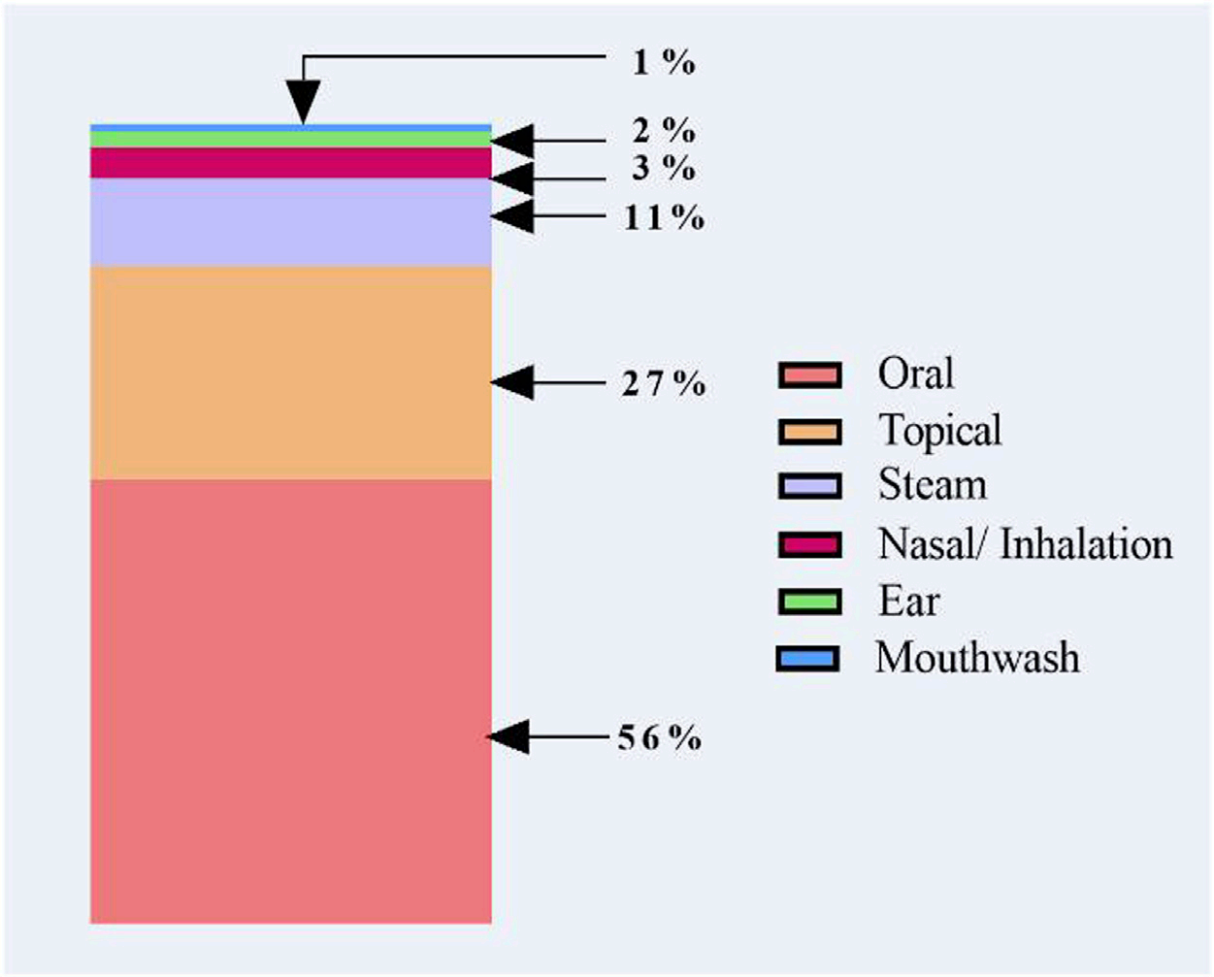
Modes of administration.

**FIGURE 6:**
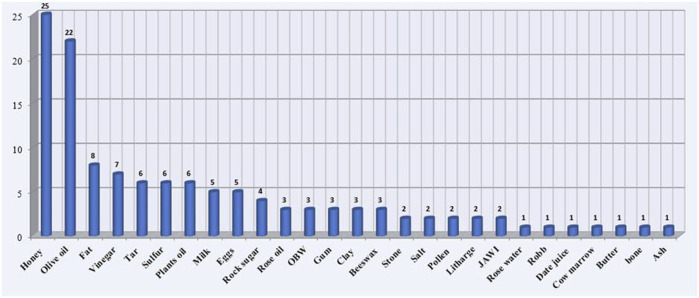
Adjuvants added.

Regarding the preparation methods (Figure 4), decoction (35%), raw (24%), infusion (19%), paste (10%), and maceration (8%) were the dominant methods for remedies preparation. Surprisingly, the current study recorded burning (2%) as an uncommon/novel mode used by traditional healers. In addition, the common administration route was the oral ingestion (56%) followed by external application as an ointment on the skin and compress (27%), steam (11%), or internally tract as nasal inhalation (3%), intraear (2%), and the mouthwash (1%) (Figure 5). Of the remedy’s prescription, 64% of medicinal plants were mixed with other ingredients, and 36% were taken without addition. Indeed, there were 32 species combined with one plant, 21 plants with two plants, 19 plants with three or four plants, and 14 plants with more than four plants. Furthermore, some herbal mixtures (43%, *n* = 74 species) were prepared by adding different adjuvants (Figure 6). These adjuvants include honey (25 use reports) followed by olive oil (22), fat (8), vinegar (7), plant oil, and sulfur and tar (6 times each).

### New Reports and New Uses

By comparing the data from this study with other ethnobotanical researches carried out in Algeria and neighboring countries (Morocco, Tunisia, Mauritania, Nigeria, and Mali), we found that 11% of total species have not been previously reported as medicinal plants. Of them, 11 species were documented in Sahara, 5 in Kabylia, and 3 in West Algeria. These species are used as remedies to treat both common ailments and incurable diseases. The new reports are listed in Table 3 with their vernacular names, parts used, therapeutic uses, and modes of administration.

**TABLE 3:** New recorded medicinal plants used by traditional healers in Algeria (West-Kabylia-Sahara).

Scientific name	Local name	Ailments	Number of informants citing plants	Number of citations
*Inula helenium* L.	مطهر	Can: 2* breast cancer and legs cancer	1	2
*Centaurea acaulis* L.	*سنتوريا او القنطريون*	Can: 2* breast cancer and legs cancer	1	2
*Melilotus officinalis* (L.) Pall.	الهندقوق إكليل الملك	RTD: 1* chest and lung diseases	1	2
		GISD: 1* IBS		
*Lupinus micranthus* Guss.	الترمز المر	ESD: 1* diabetes	1	1
	الدقيق			
*Boswellia ameero* Balf.f.	اللبان	RTD: 1* chest and lung diseases	1	1
*Carduus nutans* L.	شوك المحني	HC: 1* alopecia areata	1	1
*Quercus faginea* Lam.	العفص	SRP: 1* uterine microbe	1	1
*Gentiana acaulis* L.	كف الذئب او الجنطيانا	Can: 1* breast cancer and legs cancer	1	1
*Digitalis purpurea* L.	القِمَعية او الدِّيجيتال	CVSD: 1* cardiovascular diseases	1	1
*Cistanche tubulosa* (Schenk) Wight	ذنون	GISD: 1* colitis	1	1
*Hypecoum procumbens* L.	جهيرة (الخشخاشية)	Can: 1* skin pimples and tumors	1	1
*Phyllanthus niruri* L.	الأَمْلَج	Can: 1* cancer	1	2
		RTD: 1* cough		
*Verbascum sinuatum* L.	مصلح الأنظار أو البوصير أو تيسراو	SMSD: 1* osteoarthritis	1	1
*Lycium shawii* Roem. and Schult.	العوسج	SD: 2* skin ulcers and leprosy	2	3
		RTD: 1* pneumonia		
*Tamarix aphylla* (L.) H.Karst.	طحطاح	NS: 1* headache	1	1
*Ulmus rubra* Muhl.	الدردار	ID: 1* laryngitis	2	2
		SMSD: 1* moving difficulty		
*Telephium imperati* L.	تسمرغينت	GH: 1* mouth ulcer	1	2
		HSD: 1* anemia		
*Humulus lupulus* L.	جنجل	HC: 1* alopecia areata and baldness	2	3
		GISD: 2* hemorrhoids		
*Cirsium creticum* (Lam.) d'Urv.	شوك الرمح	GISD: 1* hemorrhoids	1	1

Surprisingly, 4 out of the 19 new species (*Lycium shawii* Roem*.* and Schult, *Humulus lupulus* L., *Crataegus azarolus* L.*, Centaurea acaulis* L., and *Verbascum sinuatum* L*.*) were highly cited by the informants. *V. sinuatum* is used to treat gastrointestinal and respiratory tract diseases such as pneumonia, using the decoction method with oral and topical application, respectively. The plant is termed locally “Moslih el-Andar” meaning in the local dialect “tract’s fixer” relating to its effect that repairs the continuous elongated anatomical structure in the body. Similarly, the decoction of *L. shawii* is used to treat two ailments categories: skin diseases (skin ulcers and leprosy) and skeletomuscular system disorder (osteoarthritis). Nevertheless, these ethnomedicinal uses and their pharmacological properties have not been documented in previous studies.

Furthermore, some species were previously reported to be used for culinary purposes such as *Telephium imperati* L. called in local dialect as tassarghit/sarghina. The stems of the plant are usually consumed as soup’ spice for postpartum women in Algeria (Sahara and Kabylia region). As reported here, for the first time, it is newly mentioned to be used for medicinal purposes by the local healers treating mouth ulcers and anemia. Moreover, we found that decoction of *Quercus faginea* Lam. seeds, a popular tree in West Algeria (Alcaraz, 1989), is used to treat sexual-reproductive problems besides the fruits (raw) of *Lupinus micranthus* Guss., a species widely distributed in Algeria and the Mediterranean countries (Msaddak et al., 2017). On the other hand, our results showed that species such as *Phyllanthus niruri* L.*, Hypecoum procumbens* L*.,* and *Gentiana acaulis* L. are used to treat skin diseases and cancer *via* topical application. These species have not been previously reported to be used as medicinal species in the Mediterranean region.

In the present study, we found that 89% of total species have already been mentioned as medicinal plants. In fact, more than 100 species cited in this survey were reported in previous studies from different regions of Algeria. Besides, we found that, despite having similar therapeutic uses, the species had different vernacular names from a region to another. Interestingly, we report here 47 new therapeutic uses for 20 known plant species. Table 4 shows these new uses compared to those previously reported in the world.

**TABLE 4:** List of new therapeutic uses recorded in Algeria (West-Kabylia-Sahara).

Botanical name	Part used	New uses	Preparation methods	Previously reported uses	References
*Silybum marianum* (L.) Gaertn.	Leaves	Can: 2 breast cancer and legs cancer	Raw	Biliary, liver disorders, and degenerative necrosis	Lahlah et al. (2012)
				Jaundice and enlarged spleen	
*Prunus persica* (L.) Batsch.	Leaves	Can: 2* cancer	Raw	Cough, constipation, and menstruation absent	Lin et al. (2021)
		Sd: 1* limb swelling	Infusion		Al-Fatimi. (2019)
*Inula helenium* L.	Capitulum	Can: 2* breast cancer and legs cancer	Raw	Hematomas, relief of bruises, joint pains, rheumatism, and gastrointestinal, otolaryngological, and respiratory diseases	Teixidor-Toneu et al. (2016)
					Obón et al. (2012)
*Calendula arvensis* M. Bieb.	Capitulum	Rtd: 1* pneumonia	Decoction	Burns, varicose veins, eczema, fungus, warts, and wounds	Lievre et al. (1992)
					Lavagna et al. (2001)
*Artemisia campestris* L.	Leaves	P: 1* scorpion sting	Raw	Digestive troubles, gastric ulcer, and menstrual pains	Baba Aissa (1991)
*Cichorium intybus* L.	Aerial part/roots	Usd: 2* cystolithiasis and bladder disease	Decoction	Urinary tract infections and urolithiasis, digestive problems, kidney diseases, diabetes, and nervous disorders	Sekkoum et al. (2011)
			Decoction and raw		Miara et al. (2013)
		Gisd: 2* hemorrhoids and liver diseases	Raw		El-Hilaly et al. (2003)
			Decoction		Daoudi et al. (2016)
					Benarba et al. (2015)
		Can: 4* breast cancer and legs cancer	Decoction		
		Hsd: 1* spleen diseases	Decoction		
*Carlina gummifera* (L.) Less.	Capitulum/leaves/roots	Srp: 2* infertility and uterine problems	Decoction	Epilepsy, psoriasis, ulcers, and hemorrhage	Bellakhdar (1997)
			Decoction		Ahid et al. (2012)
		Usd: 2* urinary tract infection and bladder disease	Decoction		Hammiche et al. (2013)
			Decoction		
		Smsd: 1* osteoarthritis	Decoction		
*Echinops spinosissimus* Turra.	Aerial part	Can: 1* skin pimples and tumors	Decoction	Hypotensive, diuretic, hypoglycemic, for stomachic effects, liver disorders, and postpartum care	Bouzabata (2013)
*Clinopodium nepeta* (L.) Kuntze.	Aerial part	Gisd: 1* IBS	Decoction	Colon ailments, abdominal pain, influenza, heart problems, bee, and insect stings	Mattalia et al. (2020)
		Esd: 1* diabetes	Decoction		Çelik et al. (2021)
		Cvsd: 1* cholesterol	Decoction		
		Kd: 1* kidney failure	Decoction		
		Usd: 1* bladder disease	Decoction		
*Mentha rotundifolia* (L.) Huds.	Aerial part	Gisd: 1* IBS	Decoction	Mental illnesses, colds, respiratory problems and to protect removal of “curses” and “evil spirits”	Arnold and Gulumian (1984) Pooley (2005)
*Potentilla erecta* (L.) Raeusch.	Roots	Srp: 1* breast milk outage	Maceration	Wounds, certain forms of cancer, infections, diarrhea, and diabetes mellitus	Synowiec et al. (2014)
		Rtd: 1* chest and lung diseases	Raw		
		Gisd: 2* stomach ache and ulcers	Maceration		
			Decoction		
*Amaranthus spinosus* L.	Aerial part	Srp: 1* infertility	Decoction	Internal bleeding, diarrhea, excessive menstruation, and snake bites. Ulcerated mouths, nosebleeds, and wounds	Saravanan (2016)
				Menorrhagia, gonorrhea, eczema and colic, fevers, and urinary troubles	
*Mahonia aquifolium* (Pursh) Nutt.	Whole plant	Can: 2* breast cancer and legs cancer	Raw	Skin diseases, psoriasis, and diabetes	Galle et al. (1994)
					Missoun et al. (2018)
*Boswellia ameero* Balf.f.	Gum	Rtd: 1* chest and lung diseases	Maceration	Antitumor activity	Shao et al. (1998)
*Commiphora myrrha* (Nees) Engl.	Wax	Can: 2* breast cancer and legs cancer	Raw	Mouth ulcers, gingivitis, sinusitis, glandular fever, brucellosis, and antiparasitic agent	Abdel-Hay et al. (2002)
			Raw	Autoimmune diseases, rheumatic pains, amenorrhea, fever, stomach complaints, gall bladder, nephrosis syndrome, chest ailments, snake and scorpion bites, mouth ulcer, and skin infections	Abdul-Ghani et al. (2009)
					Boual et al. (2020)
					El Ashry et al. (2003)
					Massoud et al. (2001)
*Cymbopogon schoenanthus* (L.) Spreng.	Leaves	Can: 1* skin pimples and tumors	Decoction	Termites and bruchid, digestive diseases, aerophagia, flatulence and urinary decrease, analeptic, bad breath, gumboils, and urinary incontinence	Koba et al. (2007)
					Hammiche and Maiza (2006)
*Daphne gnidium* L.	Leaves	Hc: 1* hair loss	Raw	Constipation and toothache, wounds, hair lice or ticks in animals hair washing and as hair tonic	Allal et al. (2019)
		Rtd: 1* sinusitis	Steaming		
*Cistanche tubulosa* (Schenk) Wight	Whole plant	Gisd: 1* colitis	Raw	For blood circulation and impotence, female infertility, lumbago, body weakness, and tonic substance	Namba (1994)
					Kobayashi et al. (1987)
*Phyllanthus niruri* L.	Leaves	Can: 1* cancer	Raw	Hepatoprotective functions	Bhattacharjee & Sil (2007)
		Rtd: 1* cough	Decoction		
*Tetraena alba* (L.f.) Beier and Thulin.	Leaves/seeds	Esd: 1* diabetes	Decoction	Diabetes, intoxication (toukal), gastrointestinal disorders, hypertension, and arteriosclerosis	Benali et al. (2017)
					Mnafghi et al. (2016)

### Informant Consensus Factor and FL

Table 5 shows the 16 ailments categories arranged in descending order based on the F_IC_ values. Cancer had the highest F_IC_ value of 0.49 with 44 species used, such as *C. colocynthis, Panax ginseng* C.A. Mey.*, E. alata, Aquilaria malaccensis, Aristolochia longa* L*.,* and *Taraxacum officinale.* On the other hand, we found that sexual-reproductive problems (F_IC_ = 0.46), gastrointestinal system diseases (F_IC_ = 0.44), and skeletomuscular system disorders (F_IC_ = 0.39) were recorded to have the second, third, and fourth highest F_IC_ values, respectively. Respiratory tract diseases were ranked to be the fifth ailment group with an F_IC_ value of 0.36.

**TABLE 5:** Informant consensus factor for commonly used medicinal.

Ailment category	Nur	Nt	F_IC_
Cancer	86	44	0.49
Sexual-reproductive problems	70	38	0.46
Gastrointestinal system diseases	100	56	0.44
Skeletomuscular system disorder	32	20	0.39
Respiratory tract diseases	51	33	0.36
Skin diseases	42	29	0.32
Urology system diseases	26	21	0.20
General health	39	32	0.18
Nervous system	36	30	0.17
Kidneys diseases	9	8	0.13
Hair care	11	8	0.30
Endocrine system diseases	12	11	0.09
Hematological system diseases	17	16	0.06
Cardiovascular system diseases	18	17	0.06
Poisoning	2	2	0.00
Infectious diseases	2	2	0.00

According to their knowledge and experience, the local healers preferred some species to treat particular diseases. The highest FL values of the commonly used plants are listed in Table 6. Our results indicated that *M. vulgare, A. herba-alba, Z. officinale,* and *J. phoenicia* had the absolute FL value of 100% in several ailment categories (SRD, cancer, respiratory diseases, and GISD).

**TABLE 6:** FL of commonly used medicinal plants.

Ailment category	Species	FL (100 (%)
KD	*Cichorium alatum* Hochst. and Steud.	100
	*Artemisia herba-alba* Asso.	50
	*Parietaria officinalis* L.	100
GISD	*Marrubium vulgare* L.	100
	*Zingiber officinale* Roscoe	100
	*Juniperus Phoenicea* L.	100
	*Artemisia herba-alba* Asso.	100
	*Matricaria chamomilla* L.	80
	*Punica granatum* L.	67
	*Rhamnus alaternus* L.	67
	*Curcuma longa* L.	67
SD	*Thymus vulgaris* L.	100
	*Origanum majorana* L.	50
	*Eruca sativa* L.	50
Can	*Carum carvi* L.	50
	*Thapsia garganica* L.	33
	*Marrubium vulgare* L.	100
	*Zingiber officinale* Roscoe	100
	*Juniperus phoenicea* L.	100
	*Artemisia herba-alba* Asso.	100
	*Matricaria chamomilla* L.	40
	*Ziziphus spina-christi* (L.) Desf.	50
ESD	*Pimpinella anisum* L.	17
	*Saccocalyx satureioides* Coss. and Durieu.	100
RTD	*Marrubium vulgare* L.	100
	*Zingiber officinale* Roscoe	100
	*Glycyrrhiza glabra* L.	67
	*Juniperus phoenicea* L.	100
	*Artemisia herba-alba* Asso.	100
	*Pinus maritima* L.	50
	*Calendula arvensis* M.Bieb.	50
SMSD	*Echinops spinosissimus* Turra.	67
	*Tussilago farfara* L.	100
	*Echinops ritro* L.	100
CVSD	*Myrtus nivellei* Batt. and Trab.	51
	*Crataegus azarolus* L.	50
GH	*Nicotiana tabacum* L.	50
	*Pistacia lentiscus* L.	53
	*Carthamus tinctorius* L.	50
HC	*Carduus nutans* L.	100
	*Daphne gnidium* L.	69
NS	*Crocus sativus* L.	53
	*Eriobotrya japonica* (Thunb.) Lindl.	67
SRP	*Asarum europaeum* L.	100
	*Hyacinthus orientalis* L.	80
	*Marrubium vulgare* L.	100
	*Zingiber officinale* Roscoe	100
	*Juniperus phoenicea* L.	100
	*Artemisia herba-alba* Asso.	100
ID	*Ulmus rubra* Muhl.	50
P	*Artemisia campestris* L.	100
HSD	*Cichorium alatum* Hochst. and Steud.	100
	*Salvia hispanica* L.	100
	*Vitis vinifera* L.	50
	*Rubia tinctorum* L.	33
USD	*Urtica dioica* L.	34
	*Nitraria retusa* (Forssk.) Asch.	50

## Discussion

### Botanical Diversity, Parts Used, Modes of Preparation, and Administration

In the present study, we recorded 167 species belonging to 70 families with a dominance of Lamiaceae, Asteraceae, Apiaceae, Rosaceae, and Fabaceae. Our findings agreed with those we previously reported. Indeed, in Mascara (North-West Algeria), most of the medicinal species used by local healers belonged to these five families (Benarba, 2015). Similar findings were reported in Algeria (Miara et al., 2018; Taibi et al., 2020), Morocco (Barkaoui et al., 2017; Skalli et al., 2019), and Italy (Tuttolomondo et al., 2014). It has been suggested that plants belonging to these families are mainly used by local populations in Africa owing to their pharmacological effects offering a cheap therapeutic alternative (Sawadogo et al., 2012). Furthermore, leaves, aerial parts, and seeds were the most frequently used parts by local healers. Our results confirm the dominance of leaves as the most common used important plants’ part used in local phytotherapy as has been demonstrated in Algeria (Benarba, 2015; Benarba, 2016; Bouasla and Bouasla 2017; Miara et al., 2018; Taibi et al., 2020) and neighboring countries such as Mauritania (Yebouk et al., 2020), Morocco (Barkaoui et al., 2017; Skalli et al., 2019), or Italy (Leto et al., 2013). The dominance of leaves in most of the ethnobotanical studies could be explained by their ease collecting and abundance besides the fact that they are considered the site of photosynthesis and therefore of bioactive molecules.

Our results showed that the traditional healers used different preparation methods, including decoction, infusion, paste, or maceration. Decoction was found to be the preferred method. Similar results were found in previous ethnobotanical studies (Benarba, 2015; Merrouni and Elachouri, 2020). In fact, decoction and infusion were found to be the most used in the recent ethnobotanical studies in Algeria (Benarba et al., 2015; Benarba, 2016; Mechaala et al., 2021; Zatout et al., 2021) and neighboring countries such as Tunisia, Egypt, Spain, and Italy in Africa and in Europe (Giday et al., 2009; Benitez et al., 2010; Amri and Kisangau, 2012; Menale et al., 2016; Savić et al., 2019). The dominance of decoction or infusion could be explained by the disinfection potential of heating besides its extraction enhancing effects (Benarba, 2015).

We also found that oral ingestion was the most frequently used mode of administration, followed by external application, steam, and nasal inhalation. Our findings are consistent with those we previously reported in South-West Algeria (Benarba, 2016), North-West Algeria (Benarba, 2015), and Extreme-West Algeria (Tlemcen) (Zatout et al., 2021). Likewise, oral and topical applications were found to be the most frequently used administration methods used by local healers or populations in other regions in Algeria (Hammiche and Maiza, 2006; Boudjelal et al., 2013; Sarri et al., 2014; Miara et al., 2018) and neighboring countries (Mrabti et al., 2019; Fakchich and Elachouri, 2014; Benitez et al., 2010). In this same line, oral and topical administrations are frequently used in traditional medicine. The choice of administration routes is based on the pharmacological effect of each species, the therapy target, duration, and the limitation of treatment to a precise area (Sargin et al., 2015; Benarba, 2016).

The traditional healers in the study areas reported that 64% of medicinal species were mixed with other medicinal plants, whereas 43% of herbal mixtures were prepared by adding different adjuvants with a dominance of honey, olive oil, animal fat, or vinegar. In concordance with our findings, several ethnobotanical investigations carried out in Algeria demonstrated that honey was the adjuvant most frequently added to prepare medicinal herbal mixtures (Benarba, 2016; Ouelbani et al., 2016; Zatout et al., 2021). Our findings are also in perfect consistency with those reported in other regions around the world (Yabesh et al., 2014; Amri and Kisangau, 2012; Pranskuniene et al., 2016). These ingredients could enhance the plant effect, maintain the blend texture, and facilitate the treatment administration. To the best of our knowledge, some adjuvants were not previously mentioned, such as tar and litharge.

### New Reports and New Uses

In the present study, 11% of the recorded 167 species have not been previously reported as medicinal plants in Algeria and neighboring countries in the Mediterranean basin. Moreover, more than 100 species reported here were previously reported to be used for therapeutic purposes in North-West (Benarba, 2015), South-West (Benarba, 2016), and North-East Algeria (Boual et al., 2020). Although each species had mostly the same therapeutic uses, for example, *A. herba-alba*, *Punica granatum* L., and *Senna alexandrina* Mill. were used mainly to treat gastrointestinal disorders, their vernacular names differed from one region to another such as *Aquilaria malaccensis* Lam. called Oud El-Rih in the West and A-ghriss in Sahara. These findings are in agreement with those reported in Algeria (Benarba, 2015; Bouasla and Bouasla, 2017 ), Morocco (Chaachouay et al., 2020; Merrouni and Elachouri, 2020; Yebouk et al., 2020), and other countries such as Yamen, Turkey, India, and China (Prabhu et al., 2014; Polat, 2019).

Interestingly, our findings report 47 new therapeutic uses for 20 known plant species. In the present study, we found that local populations living in the study areas used *Carlina gummifera* (L.) Less. to treat infertility, uterine problems, urinary tract infection, bladder disease, and osteoarthritis, whereas the plant uses previously reported included epilepsy, psoriasis, ulcers, and hemorrhage (Ahid et al., 2012; Hammiche et al., 2013). Likewise, leaves of *Cymbopogon schoenanthus* (L.) Spreng. were found to be used in the treatment of several types of cancer in the study areas. This use is reported for the first time since the plant was previously reported to be used mainly to treat termites and bruchid (Koba et al., 2007). *Prunus persica*, usually used against cough, constipation, and menstruation absent (Lin et al., 2021; Al-Fatimi., 2019), was reported by local populations to treat skin diseases.

### Informant Consensus Factor and FL

Regarding the informant consensus factor, the highest F_IC_ value was recorded for cancer (F_IC_ = 0.49) with 44 medicinal species used. This is the first study carried out in the three regions (West, Sahara, and Kabylia) of Algeria at the same time, calculating the informant consensus factor (F_IC_). Our results revealed that cancer seems to be one of the most prevalent diseases in the study areas since no previous investigations had found cancer as the first ailment category according to their F_IC_ values. In fact, cancer has become a public health issue due to an increasing incidence, with 19.3 million new cases and about 10.0 million deaths worldwide in 2020 (Ferlay et al., 2021). Likewise, cancer incidence is increasing in Algeria. Actually, Algeria has the highest incidence of gastric (6%) (Behar et al., 2021) and liver cancer (Benarba and Meddah, 2014) when compared to North African countries. Moreover, breast and thyroid cancer incidence rose significantly in the last two decades (Mehemmai et al., 2020; Halfaoui et al., 2021). This pattern may be attributed to several causes, such as a westernized lifestyle, contaminated foods, pollution, and deteriorated living conditions. Furthermore, sexual-reproductive problems, gastrointestinal system diseases, skeletomuscular system disorders, and respiratory tract diseases were recorded to have higher F_IC_ values. In a previous study carried out in North-West Algeria, we found that gastrointestinal diseases had the highest F_IC_ value of 0.658, followed by general health (F_IC_ = 0.645) and respiratory diseases (0.642), while the cancer category was recorded to be the 4th highest (F_IC_ = 0.524) (Benarba et al., 2015). Moreover, a recent study carried out in the extreme North-West of Algeria reported that the reproductive and sexual disorders F_IC_ value were the highest score (0.98), and for the cancer category, they had an F_IC_ value of 0.77 with 6 species (Zatout et al., 2021). In disagreement with our findings, Bouasla and Bouasla (2017) indicated that cancer (F_IC_ = 0.25) was the least known ailment to be treated in the traditional medicine of the local population in North-East Algeria.

According to our results, *M. vulgare, A. herba-alba, Z. officinale,* and *J. phoenicia* had the absolute FL value of 100% in several ailment categories (SRD, cancer, respiratory diseases, and GISD). These findings are in agreement with those previously reported in different neighboring regions (Benarba et al., 2015; Bouasla and Bouasla, 2017; Chaachouay et al., 2020). Besides these species, *Parietaria officinalis* L. was found to possess an FL of 100% for kidney diseases which is consistent with findings previously reported in North-West Algeria (Benarba, 2016) and Morocco (Ammor et al., 2020). Inconsistent with our previous findings in both North-West (Benarba et al., 2015) and South-West Algeria (Benarba, 2016), *T. vulgaris* was the only species having the highest FL of 100% for skin diseases. This could be attributed to its antifungal and antimicrobial potentials demonstrated against the main pathogens causing skin diseases (Tadele et al., 2009; Vinciguerra et al., 2019). Recently, a facial phytocosmetic preparation from *T. vulgaris* was found to possess promising antiskin aging effects, as shown by enhanced adipogenesis through upregulation of PPAR-γ expression (Caverzan et al., 2021).

## Conclusion

This is the first study carried out in three regions in Algeria (North, Center, and South) revealing an important botanical diversity and ethnobotanical knowledge held by local populations. The ethnobotanical survey allowed us to document 167 medicinal plants belonging to 70 families with their indigenous therapeutic uses (Table 7). Furthermore, 47 therapeutic uses for 20 known plant species were newly recorded, besides 25 species reported for the first time as medicinal plants in this study. On the other hand, *A. sativum, T. foenum-graecum, Z. officinale, R. chalepensis, A. herba-alba, P. anisum, M. chamomilla, O. basilicum,* and *T. vulgaris* had the highest UV. Moreover, some species had the absolute FL value of 100% in several ailment categories such as *M. vulgare*. These species could be further investigated to explore their curative proprieties and identify the possible active compounds.

**TABLE 7:** List of medicinal plants used by traditional healers in the study areas.

Family	Scientific name (voucher number)	Local name	Part used	Ailments	Preparation methods	Administration
Amaranthaceae	*Amaranthus spinosus* L. (LRSBG/AB/20/067)	القُطَيفة سالف العروس	Aerial part	SRP: 1* infertility	Decoction	Oral/topical
	*Haloxylon salicornicum* (Moq.) Bunge ex Boiss. (LRSBG/AB/20/068)	رمث الاحمر او تاسّايت	Leaves	Can: 1* cancer	Raw	Oral
				P: 1* poisoning	Decoction	Oral
	*Atriplex halimus* L. (LRSBG/AB/20/069)	القطف او السرمق ،الملوخ	Leaves	Can: 3* uterine cysts and tumors	Decoction	Oral
				Breast cysts and tumors	Decoction	Oral
				Cancer	Maceration	Oral
Amaryllidaceae	*Allium sativum* L. (LRSBG/AB/20/072)	الثوم	Bulb	RTD: 5* asthma	Decoction	Topical
				Chest and lung diseases	Decoction	Topical
				Cough	Raw	Topical
				Nasal-lung inflammation	Maceration/decoction	Topical/oral
						Oral
				SRP: 2* infertility	Decoction	Inhalation
				GH: 2* tonsillitis	Decoction	Topical
				Earache and deafness	Decoction	Topical
				GISD: 2* jaundice/icterus	Raw	Topical
				Liver diseases	Raw	Topical
				HC: 1* alopecia areata	Frying	Topical
				SD: 1* boils	Frying	Topical
				Can: 1* skin pimples and tumors	Decoction	Oral
	*Allium cepa* L. (LRSBG/AB/20/073)	بصل	Bulb	SRP: 3* infertility + uterine problems	Decoction	Topical
						Topical
					Decoction	Topical
				SD: 2* boils and head ulcers	Frying	Topical
					Frying	Topical
				Can: 1* skin pimples and tumors	Raw	Topical
				GISD: 1* jaundice/icterus	Raw	Topical
Anacardiaceae	*Pistacia lentiscus* L*.* (LRSBG/AB/20/123)	المَصطَكى أو المستكة او الضرو	Leaves/wax	RTD: 1* pulmonary-breathing problem	Raw	Oral
				GISD: 4* heartburn	Decoction	Oral
				stomach ache	Decoction	Oral
				Diarrhea	Decoction/raw	Oral
				GH: 2* mouth ulcer	Decoction	Oral
				Earache and deafness	Decoction	Topical
Apiaceae	*Ferula assa-foetida* L. (LRSBG/AB/20/145)	الكَلَخ او الحِلْتِيت	Whole plant	GH: 2* tonsillitis	Infusion/raw	Topical
					Raw/decoction	Oral/topical
	*Cuminum cyminum* L. (LRSBG/AB/20/001)	الكمون	Seeds	SMSD: 1* arthritis	Infusion	Topical/oral
				SRP: 2* infertility	Raw/decoction	Topical
				GISD: 2* stomach ache	Maceration	Oral
				Jaundice/icterus	Decoction	Oral
	*Ammoides pusilla* (Brot.) Breistr. (LRSBG/AB/20/002)	النوخة أو النانخة	Aerial part	GH: 1* fever	Decoction	Oral/topical
				HSD: 1* jaundice/icterus	Decoction	Oral
	*Pimpinella anisum* L. (LRSBG/AB/20/003)	حبة الحلاوة أو اليانسون	Fruits	ESD: 1* diabetes	Decoction	Oral
				CVSD: 1* cholesterol	Decoction	Oral
				KD: 1* kidney failure	Decoction	Oral
				USD: 1* bladder disease	Decoction	Oral
				RTD: 1* asthma	Decoction	Topical
				GISD: 1* stomach ache	Maceration	Oral
				NS: 1* insomnia	Decoction	Oral
				SD: 1* skin disease	Infusion	Topical
				SMSD: 2* arthritis	Infusion	Oral/topical
				SRP: 1* infertility	Decoction	Topical
	Coriandrum sativum L. (LRSBG/AB/20/004)	القزبر أو الكُـزْبـَرَة	Aerial part	SD: 2* limb swelling	Infusion	Topical
				RTD: 1* chest and lung diseases	Infusion	Topical
	*Foeniculum vulgare* Mill. (LRSBG/AB/20/005)	البسباس	Seeds	GISD: 3* IBS	Infusion	Oral
				stomach ache	Maceration	Oral
				Flatulence	Decoction	Oral
				NS: 1* headache	Infusion	Oral
	*Bunium mauritanicum* L. (LRSBG/AB/20/006)	تالغودة او آ أكثار	Roots/seeds	ESD: 1* goiter	Raw	Oral
	*Carum carvi* L. (LRSBG/AB/20/007)	كرويا	Seeds	Can: 1* early stage cancer	Decoction	Raw
				GISD: 1* stomach ache	Maceration	Oral
				GH: 1* anxiety disorders and hypochondria	Raw	Topical
				SMSD: 1* bones pain	Raw	Oral
	*Apium graveolens* L. (LRSBG/AB/20/008)	الكرفس	Leaves	SMSD: 2* osteoarthritis	Decoction	Oral/topical
	*Thapsia garganica* L. (LRSBG/AB/20/009)	درياس أو بونافع	Aerial part	RTD: 2* chest and lung diseases	Maceration/frying	Topical/oral
				Can: 1* lung tumors	Raw	Topical
	*Petroselinum crispum* (Mill.) Fuss. (LRSBG/AB/20/010)	البقدونس أو المعدَنوس	Aerial part	KD: 1* urolithiasis	Decoction	Oral
				GH: 1* mouth ulcer	Decoction	Oral
Apocynaceae	*Nerium oleander* L. (LRSBG/AB/20/109)	الدفلة	Leaves	GH: 1* mouth ulcer	Decoction	Topical
				Can: 1* skin pimples and tumors	Burned	Topical
				SD: 3* chalazion	Decoction	Topical
				Tinea capitis and scalp ringworm	Raw	Topical
				Urticaria	Maceration	Topical
Araliaceae	*Panax ginseng* C.A.Mey. (LRSBG/AB/20/124)	الجنسنغ أو الجنسة	Aerial part	Can: 1* stomach cancer	Maceration	Oral
				SRP: 1* infertility	Raw	Oral
				GISD: 1* liver diseases	Decoction	Oral
				HSD: 1* spleen diseases	Decoction	Oral
Aristolochiaceae	*Asarum europaeum* L. (LRSBG/AB/20/096)	أسارون	Leaves	SRP: 1* uterine microbe and infections	Decoction	Oral
	*Aristolochia longa* L. (LRSBG/AB/20/097)	برسطم—برزطم	Stalk	HC: 1* baldness	Raw	Topical
				Can: 3* breast cancer	Raw	Topical
				Legs cancer	Raw	Topical
				Cancer	Raw	Oral
Asparagaceae	*Hyacinthus orientalis* L. (LRSBG/AB/20/098)	الخُزَامَى	Flowers	SRP: 4* infertility	Raw/decoction	Topical
				Uterine problems	Decoction	Topical
				USD: 2* urinary tract infection/inflammation	Decoction	Oral
				Bladder disease	Maceration	Oral
				GH: 1* fever	Decoction	Oral/topical
	*Drimia maritima* (L.) Stearn. (LRSBG/AB/20/099)	البَصَل البَرِّيّ أو بصل الحلوف	Bulb	SRP: 2* infertility	Decoction	Topical
				Uterine problems	Decoction	Topical
				HC: 1* alopecia areata	Decoction	Topical
Asteraceae	*Cynara scolymus* L. (LRSBG/AB/20/156)	العسلوج او ساق الخرشوف	Stalk	GISD: 1* hemorrhoids	Decoction	Oral
	*Arctium atlanticum* (Pomel) H.Lindb. (LRSBG/AB/20/119)	الأرقطيون	Leaves/capitulum	SD: 1* boils	Frying	Topical
				Can: 1* skin pimples and tumors	Frying	Topical
	*Cirsium creticum* (Lam.) d'Urv. (LRSBG/AB/20/160)			GISD: 1* hemorrhoids	Decoction	Topical/oral
	*Carthamus tinctorius* L. (LRSBG/AB/20/011)	العُصْفُر أو الجُرْجُوم	Capitulum	GH: 1* anxiety disorders and hypochondria	Raw	Oral/topical
	*Dittrichia viscosa* (L.) Greuter	مقرمان	Aerial part	SD: 2* festering wounds	Maceration/raw	Oral/topical
	(LRSBG/AB/20/012)			Skin diseases	Maceration	Topical
	*Tussilago farfara* L. (LRSBG/AB/20/013)	حشيشة السعال أو تافيفرا	Aerial part	SMSD: 2* osteoarthritis	Decoction	Oral/topical
	*Echinops ritro* L*.* (LRSBG/AB/20/014)	تاسكرا أو الشوك الأزرق ، و أبونقار	Aerial part	SMSD: 2* osteoarthritis	Decoction	Oral/topical
	*Saussurea costus* (Falc.) Lipsch. (LRSBG/AB/20/015)	القسط الهندي	Roots	SRP: 1* infertility	Infusion	Topical
	*Silybum marianum* (L.) Gaertn. (LRSBG/AB/20/016)	الخرفيش	Leaves	Can: 2* breast cancer	Raw	Topical
				Legs cancer		
	*Centaurea acaulis* L. (LRSBG/AB/20/017)	سنتوريا او القنطريون	Aerial part	Can: 4* breast cancer	Raw	Topical
				Legs cancer		
				Can: 4* tumors and skin pimples		
	*Inula helenium* L. (LRSBG/AB/20/018)	المطهر	Capitulum	Can: 2* breast cancer	Raw	Topical
				Legs cancer		
	*Calendula arvensis* M.Bieb. (LRSBG/AB/20/019)	عين البقر	Capitulum	RTD: 1* pneumonia	Decoction	Oral
	*Artemisia campestris* L. (LRSBG/AB/20/020)	التگوفت	Leaves	P: 1* scorpion sting	Raw	Topical
	*Anacyclus valentinus* L. (LRSBG/AB/20/021)	القرطوفة	Aerial part	HSD: 1* anemia	Decoction	Oral
	*Taraxacum officinale* (L.) Weber ex F.H.Wigg. (LRSBG/AB/20/022)	هِنْدَبَاءُ البَرِ، اليعصيب	Aerial part	Can: 2* breast cancer	Raw	Topical
				Legs cancer		
	*Anacyclus pyrethrum* (L.) Lag. (LRSBG/AB/20/023)	تيقنطيست أوعاقر قرحا	Leaves	RTD: 1* pulmonary-breathing problem	Raw	Topical
				SRP: 1 infertility	Raw	Oral
				SMSD: 2* arthritis	Maceration	Topical and oral
	*Cichorium alatum* Hochst. and Steud. (LRSBG/AB/20/024)	تمرزوق. العلت	Aerial part/roots	USD: 2* cystolithiasis	Decoction	Oral
				Bladder disease	Decoction	Oral
				GISD: 2* hemorrhoids liver diseases	Raw	Topical
					Raw	Topical
				Can: 4* breast cancer	Decoction	Oral
				Legs cancer	Decoction	Oral
				KD: 1* urolithiasis	Decoction	Oral
				HSD: 1* spleen diseases	Decoction	Oral
	*Matricaria chamomilla* L. (LRSBG/AB/20/025)	البابونج	Capitulum	GISD: 4* liver diseases	Decoction	Oral
				IBS stomach ache	Decoction	Oral
				Heartburn	Raw	Topical
					Raw	Topical
				HSD: 1* spleen diseases	Raw	Topical
				SD: 1* skin ulcers	Decoction	Oral
				Can: 2* breast cancer	Decoction	Oral
				Legs cancer	Decoction	Oral
				USD: 2* urinary tract infection/inflammation	Decoction	Oral
				Bladder diseases	Infusion	Oral
				NS: 1* insomnia	Infusion	Oral
	*Carlina gummifera* (L.) Less. (LRSBG/AB/20/026)	الأداد	Capitulum leaves/roots	SRP: 2* infertility	Decoction	Topical
				Uterine problems	Decoction	Topical
				USD: 2* urinary tract infection and bladder disease	Decoction	Topical
					Decoction	Topical
				SMSD: 1* osteoarthritis	Decoction	Oral/topical
	*Echinops spinosissimus* Turra (LRSBG/AB/20/027)	شوك الجمل	Aerial part	Can: 1* skin pimples and tumors	Decoction	Oral
	*Artemisia herba-alba* Asso. (LRSBG/AB/20/028)	الشيح	Aerial part	GH: 1* tonsillitis	Infusion	Topical
				Can: 2* skin cancer	Raw	Topical
				Breast cancer	Maceration	Oral
				ESD: 2* diabetes	Decoction	Oral
				CVSD: 1* cholesterol	Decoction	Oral
				KD: 1* kidney failure	Decoction	Oral
				USD: 1* bladder disease	Decoction	Topical
				RTD: * asthma	Decoction	Topical/oral
				GISD: 2* IBS and liver diseases	Decoction	Topical
				SRP: 2* infertility and uterine problems	Decoction	Topical
	*Carduus nutans* L. (LRSBG/AB/20/104)	شوك المحني	Capitulum	HC: 1* alopecia areata	Raw	Topical
Berberidaceae	*Berberis vulgaris* L. (LRSBG/AB/20/070)	عود الريح	Roots/bark	SRP: 1* infertility	Decoction	Topical
	*Mahonia aquifolium* (Pursh) Nutt. (LRSBG/AB/20/071)	اريغون	Whole plant	Can: 2* breast cancer and legs cancer	Raw	Topical
Betulaceae	*Betula pendula Roth* (LRSBG/AB/20/149)	عصير الشجر (الباتولية)	Bark	GISD: 1* ulcers	Infusion	Oral
Boraginaceae	*Borago officinalis* L. (LRSBG/AB/20/165)	عشبة الثور	Aerial part	SRP: 1* infertility	Decoction	Oral
Brassicaceae	*Armoracia rusticana* P.Gaertn., B.Mey. and Scherb. (LRSBG/AB/20/085)	فجل العود او الخيل	Aerial part	GH: 2* mouth ulcer	Decoction	Topical
				Halitosis	Decoction	Topical
	*Sinapis arvensis* L. (LRSBG/AB/20/086)	الخردل	Seeds	RTD: 1* chest and lung diseases	Decoction	Inhalation
	*Eruca sativa* Mill. (LRSBG/AB/20/087)	الكثأ أو الجرجير	Aerial part	SD: 1* boils	Frying	Topical
				Can: 1* skin pimples and tumors	Frying	Topical
	*Lepidium sativum* L. (LRSBG/AB/20/088)	حب الرشاد أو الحبة الحمراء الحرف	Seeds	SRP: 1* breast milk outage	Maceration	Oral
				RTD: 1* chest and lung diseases	Raw	Oral
				Can: 1* cancer	Raw	Oral
				GISD: 2* colitis	Raw	Oral
				Flatulence	Raw	Oral
	*Anastatica hierochuntica* L. (LRSBG/AB/20/154)	عشبة مريم	Leaves	GISD: 1* gastrointestinal diseases	Decoction	Oral
Burseraceae	*Boswellia ameero* Balf.f. (LRSBG/AB/20/074)	اللبان	Resin	RTD: 1* chest and lung diseases	Maceration	Topical
	*Commiphora myrrha* (Nees) Engl. (LRSBG/AB/20/075)	المر	Wax	Can: 2* breast cancer	Raw	Topical
				Legs cancer	Raw	Topical
Cactaceae	*Opuntia ficus-indica* (L.) Mill. (LRSBG/AB/20/126)	التين الشوكي الكرموس	Leaves	GISD: 1* liver diseases	Maceration	Oral
				NS: 2* headache and dizziness	Decoction	Oral
Cannabaceae	*Humulus lupulus* L. (LRSBG/AB/20/153)	جنجل	Leaves	HC: 3* alopecia areata	Raw	Topical
				Baldness		
				NS: 1* headache	Raw	Topical
				GISD: 2* hemorrhoids	Raw	Topical
				ID: 2* mouth and ears infections	Raw	Topical
Cucurbitaceae	*Cucurbita maxima* Duchesne (LRSBG/AB/20/100)	القرع البلدي	Seeds	NS: 1* migraine	Decoction	Inhalation
	*Citrullus colocynthis* (L.) Schrad (LRSBG/AB/20/101)	الحنظل	Fruits	SD: 1* skin ulcers and leprosy	Decoction	Oral
				GISD: 1* constipation	Decoction	Oral
				Can: skin cancer	Maceration	Oral/topical
Cupressaceae	*Juniperus foetidissima* Willd. (LRSBG/AB/20/089)	العَرعَر	Aerial part	GISD: 5* IBS and stomach ache	Decoction	Oral
				Heartburn	Decoction	Oral
					Decoction	Oral
				RTD: 2* chest and lung diseases	Raw	Oral
				Can: 5* breast cancer	Raw	Topical
				Legs cancer	Raw	Topical
				SD: 1* urticaria	Maceration	Topical
				SRP: 5* infertility	Decoction	Topical
	*Cupressus sempervirens* L. (LRSBG/AB/20/090)	السرو	Leaves	USD: 1* bladder disease	Decoction	Oral
				SMSD: 1* arthritis	Infusion	Topical
Cyperaceae	*Cyperus esculentus* L. (LRSBG/AB/20/111)	حب عزيز	Seeds	HSD: 1* anemia	Infusion	Oral
Ephedraceae	*Ephedra alata Decne.* (LRSBG/AB/20/127)	العلندى	Aerial part	Can: 1* breast cysts and breast tumors	Raw	Topical
Equisetaceae	*Equisetum arvense* L. (LRSBG/AB/20/128)	ذيل الحصان ، وذنب الخيل	Aerial part	SMSD: 1* arthritis	Decoction	Oral
Fabaceae	*Ceratonia siliqua* L. (LRSBG/AB/20/162)	الخَرّوب	Seeds	GISD: 1* gastrointestinal diseases	Raw	Oral
	*Glycyrrhiza glabra* L. (LRSBG/AB/20/029)	العرقسوس	Roots	RTD: 2* cough	Decoction	Oral
				Lung filtering/smoker	Infusion	Oral
				HSD: 1* spleen diseases	Decoction	Oral
				NS: 1* head problems	Decoction	Oral
				Psychosis	Raw	Topical
	*Senna alexandrina* Mill. (LRSBG/AB/20/030)	السنامكي	Leaves	SD: 1* skin diseases	Maceration	Topical
				GISD: 4* colitis	Infusion/decoction	Oral
				Flatulence		
				IBS		
				Constipation		
				NS: 1* head problems	Decoction	Topical
				Psychosis		Oral
	*Acacia senegal* (L.) Willd. (LRSBG/AB/20/031)	الصمغ العربي	Gum	SD: 1* lichen	Infusion	Topical
	*Acacia gummifera* Willd. (LRSBG/AB/20/032)	أُمّ غَيْلاَن	Leaves	Can: 3* cancer, stomach cancer, and liver cancer	Decoction	Oral
				GH: 1* incurable diseases	Decoction	Oral
	*Trigonella foenum-graecum* L. (LRSBG/AB/20/033)	الحُلْبَة	Seeds	HSD: 1* anemia	Maceration	Oral
				Can: 5* breast cancer	Raw	Topical
				Legs cancer	Raw	Topical
				Cancer	Raw	Topical
				Skin pimples	Maceration	Oral
				Tumors	Raw	Oral
				SMSD: 2* fracture back pain	Raw	Topical
				GISD: 1* stomach ache	Raw	Oral
				RTD: 1* chest and lung diseases	Decoction/raw	Oral/topical
				SRP: 2* infertility	Infusion	Topical
				GH: 1* anxiety disorders and hypochondria	Raw	Topical
	*Melilotus officinalis* (L.) Pall. (LRSBG/AB/20/034)	الهندقوق إكليل الملك	Aerial part	RTD: 1* chest and lung diseases	Infusion	Topical
				GISD: 1* IBS	Decoction	Oral
	*Lupinus micranthus* Guss. (LRSBG/AB/20/035)	الترمز المر	Fruits	SRP: 1* infertility	Raw	Oral
		الدقيق				
Fagaceae	*Quercus faginea* Lam. (LRSBG/AB/20/110)	العفص	Seeds	SRP: 1* uterine microbe	Decoction	Topical/oral
Gentianaceae	*Gentiana acaulis* L. (LRSBG/AB/20/112)	كف الذئب او الجنطيانا	Leaves/flowers	Can: 1* breast cancer	Raw	Topical
				Legs cancer		
Iridaceae	*Crocus sativus* L. (LRSBG/AB/20/113)	الزعفران	Stamen	SD: 1* albinism	Raw	Topical
				NS: 1* headache	Raw	Topical
Juglandaceae	*Juglans regia* L. (LRSBG/AB/20/159)	الديرم	Aerial part/park	GISD: 1* gallstones	Decoction	Oral
Lamiaceae	*Lavandula angustifolia* Mill. (LRSBG/AB/20/163)	ضرم الحار	Aerial parts	GISD: 1* hemorrhoids	Raw	Topical
	*Mentha pulegium* L. (LRSBG/AB/20/036)	النَّعْنَاعُ الأُورُوبِيُّ او الفليو	Aerial part	RTD: 1* chest and lung diseases	Decoction	Oral
				USD: 1* urinary tract infection/inflammation	Decoction	Oral
				SRP: 1* infertility	Decoction	Oral/topical
				NS: 1* insomnia	Decoction	Oral
	*Saccocalyx satureioides* Coss. and Durieu (LRSBG/AB/20/037)	يزير البل	Leaves	GISD: 1* stomach ache	Decoction	Oral
				ESD: 2* diabetes	Infusion	Oral
	*Thymus capitatus* (L.) Hoffmanns. and Link. (LRSBG/AB/20/038)	صعتر أو الزعتر	Aerial part	SD: 1* burns	Frying	Topical
	*Mentha arvensis* L. (LRSBG/AB/20/039)	النَّعْنَاُع	Aerial part	CVSD: 1* cardiovascular diseases	Raw	Oral
				SRP: 1* infertility	Raw	Topical
				RTD: 1* chest and lung diseases	Raw	Oral
				GISD: 1* IBS	Decoction	Oral
				GH: 1* anxiety disorders and hypochondria	Raw	Oral
				NS: 2* head problems	Raw	Oral
				Psychosis insomnia	Decoction	Oral
	*Ocimum basilicum* L. (LRSBG/AB/20/040)	الريحان	Leaves	SRP: 4* infertility	Raw/decoction	Topical
				Uterine problems	Decoction	Topical
				GISD: 1* IBS	Decoction	Oral
				USD: 2* urinary tract infection/inflammation	Decoction	Oral
				Bladder disease	Infusion	Oral
				GH: 2* fever	Decoction	Oral/topical
				NS: 1* dizziness	Maceration	Topical
				CVSD: 1* hypertension	Infusion	Topical
	*Melissa officinalis* L. (LRSBG/AB/20/041)	مليسا	Leaves	NS: 1* insomnia	Decoction	Oral
	*Rosmarinus officinalis* L*.* (LRSBG/AB/20/042)	إكليل الجبل	Aerial part	CVSD: 1* cholesterol	Decoction	Oral
				GISD: 2* IBS jaundice/icterus	Decoction	Oral
					Infusion	Oral
	*Origanum majorana* L*.* (LRSBG/AB/20/043)	المَرْدَقُوشُ	Aerial part	SD: 1* limb swelling	Maceration	Topical
	*Clinopodium nepeta* (L.) Kuntze. (LRSBG/AB/20/044)	النَّابِطَة أو الفُوذَنْج الجَبَلِيّ	Aerial part	GISD: 1* IBS	Decoction	Oral
				ESD: 1* diabetes	Decoction	Oral
				CVSD: 1* cholesterol	Decoction	Oral
				KD: 1* kidney failure	Decoction	Oral
				USD: 1* bladder disease	Decoction	Oral
	*Lavandula stoechas* L. (LRSBG/AB/20/045)	الحلحال أو أسنان داود	Aerial part	ESD: 1* diabetes	Decoction	Oral
				CVSD: 1* cholesterol	Decoction	Oral
				KD: 1* kidney failure	Decoction	Oral
				USD: 1* bladder diseases	Decoction	Oral
				HSD: 1* blood purify	Decoction	Oral
	*Thymus vulgaris* L*.* (LRSBG/AB/20/046)	الزعتر البري	Aerial part	GISD: 1* IBS jaundice/icterus	Decoction	Oral
					Decoction/raw	Topical
				SRP: 2* infertility	Raw	Topical
				HC: 1* baldness	Raw	Topical
				Can: 1* breast cancer legs cancer	Raw	Topical
				USD: 1* urinary tract infection	Decoction	Oral
				GH: 1* fever	Decoction	Oral/topical
				SD: 4* skin diseases and ulcer	Decoction	Topical
					Infusion	Oral
	*Salvia officinalis* L. (LRSBG/AB/20/047)	المَرِيمِيَّةُ او القصعين المخزني	Leaves	CVSD: 1* cholesterol	Decoction	Oral
	*Salvia hispanica* L. (LRSBG/AB/20/048)	بذور شيا	Seeds	HSD: 1* anemia	Raw	Oral
	*Teucrium spinosum* L. (LRSBG/AB/20/049)	الجَعْدَةُ	Aerial part	HSD: 1* blood purify	Maceration	Oral
				GISD: 1* ulcers	Raw	Topical
				CVSD: 1* diabetes	Decoction	Oral
				RTD: 1* chest and lung diseases	Raw	Topical
	*Mentha aquatica* L. (LRSBG/AB/20/050)	حبق الماء	Aerial part	GH: 1* anxiety disorders and hypochondria	Raw	Oral
	*Marrubium vulgare* L. (LRSBG/AB/20/051)	المريوت	Aerial part	Can: 3* skin pimples and tumors	Raw	Topical
				Skin cancer	Raw	Topical
				Breast cancer	Decoction/raw	Topical/oral
				SRP: 4* infertility	Decoction	Topical
				Uterine problems	Decoction	Topical
				RTD: 1* pulmonary-Breathing problem	Frying/decoction	inhalation/topical
	*Vitex agnus-castus* L. (LRSBG/AB/20/052)	كف مريم	Leaves	Can: 3* breast tumor	Decoction	Oral
				Uterus tumor	Decoction	Oral
				Gum tumor	Decoction	Oral
				NS: 1* sciatica	Raw	Oral
	*Ajuga iva* (L.) Schreb. (LRSBG/AB/20/053)	الشندقورة	Leaves	CVSD: 1* cholesterol	Infusion	Oral
	*Teucrium polium* L. (LRSBG/AB/20/054)	خياطة الجراح	Aerial part	GISD: 1* ulcers	Raw	Oral
	*Mentha rotundifolia* (L.) Huds	تيمرصاد	Aerial part	GISD: 1* IBS	Decoction	Oral
	(LRSBG/AB/20/055)					
Lauraceae	*Cinnamomum camphora* (L.) J.Presl. (LRSBG/AB/20/076)	الكافور	Wax	NS: 1* migraine	Infusion	Topical
	*Cinnamomum verum* J.Presl (LRSBG/AB/20/077)	قرفة	Bark	NS: 2* migraine	Raw	Oral/topical
				USD: 1* urinary tract infection/inflammation	Decoction	Oral
	*Laurus nobilis* L. (LRSBG/AB/20/078)	الرند	Leaves	GISD: 1* ulcers	Decoction	Oral
Linaceae	*Linum usitatissimum* L. (LRSBG/AB/20/130)	زريعة الكتان	Seeds	GISD: 1* IBS	Decoction	Oral
				RTD: 1* chest and lung diseases	Maceration	Topical
				ESD: 1* goiter	Raw	Oral
				GH: 1* hoarseness and sore throat	Raw	Oral
				Can: 1* Skin pimples and tumors	Decoction	Topical
Lythraceae	*Lawsonia inermis* L. (LRSBG/AB/20/079)	الحناء	Leaves	Can: 1* Skin pimples and tumors	Decoction	Topical
				SMSD: 1* fracture	Raw	Topical
				SD: 3* urticaria	Maceration	Topical
				Warts	Burned	Topical
				Head ulcers	Raw	Topical
				GH: 1* anxiety disorders and hypochondria	Raw	Topical
Malvaceae	*Hibiscus sabdariffa* L. (LRSBG/AB/20/131)	كركدية. او الورد الحر	Flowers	CVSD: 1* hypertension	Infusion	Oral
Moraceae	*Ficus carica* L. (LRSBG/AB/20/155)	التين	Fruits	RTD: 2* chest and lung diseases	Decoction	Oral
				Cough		
				GISD: 3* jaundice/icterus liver diseases	Raw/infusion	Oral
					Maceration	Oral
Moringaceae	*Moringa oleifera* Lam. (LRSBG/AB/20/132)	المورينجا. او عشبة الحياة	Whole plant	GH: 1* incurable diseases	Decoction	Oral
				GISD: 1* IBS	Infusion	Oral
Myristicaceae	*Myristica fragrans* Houtt. (LRSBG/AB/20/114)	جَوزة الطَيب	Seeds	NS: 1* head problems	Raw	Topical
				Psychosis		
Myrtaceae	*Myrtus communis* L. (LRSBG/AB/20/151)	القمام	Leaves	RTD: 2* chest and lung diseases	Infusion	Topical
	*Myrtus nivellei* Batt. and Trab. (LRSBG/AB/20/167)	قمام الصحرا	Leaves	CVSD: 1* clogged arteries	Decoction	Oral
	*Syzygium aromaticum* (L.) Merr. and L.M.Perry. (LRSBG/AB/20/081)	القرنفل	Flower buds	NS: 1* migraine	Raw	Topical
				SRP: 3* infertility	Decoction/raw	Topical/oral
				RTD: 1* chest and lung diseases	Raw	Oral
				USD: 1* urinary tract infection/inflammation	Decoction	Oral
				GH: 1* earache and deafness	Decoction	Topical
				SD: 1* skin diseases, ulcer	Infusion	Topical
	*Eucalyptus globulus* Labill. (LRSBG/AB/20/082)	كَالِبتوس	Leaves	SRP: 1* infertility	Decoction	Topical
Nitrariaceae	*Nitraria retusa* (Forssk.) Asch. (LRSBG/AB/20/161)	شجرة ليهود	Leaves	USD: 1* bladder disease	Infusion	Oral
				Can: 1* tumors	Infusion	Oral
	*Peganum harmala* L. (LRSBG/AB/20/133)	الحرمل	Seeds	GISD: 1* IBS	Decoction	Oral
				SRP: 1* infertility	Raw	Topical
				RTD: 2* chest and lung diseases	Raw	Oral
				Nasal-lung inflammation	Decoction	Inhalation
				GH: 1* fever	Decoction	Oral
				USD: 1* urinary tract infection/inflammation	Decoction	Oral
Oleaceae	*Olea oleaster* Hoffmanns. and Link (LRSBG/AB/20/094)	الزبوج	Leaves	GH: 1* mouth ulcer	Decoction	Topical
	*Olea europaea* L. (LRSBG/AB/20/095)	الزيتون	Leaves fruits	GH: 1* mouth ulcer and halitosis	Decoction	Topical
				NS: 1* head problems	Raw	Topical
				Psychosis		
Orobanchaceae	*Cistanche tubulosa* (Schenk) Wight. (LRSBG/AB/20/134)	ذنون	Whole plant	GISD: 1* colitis	Raw	Oral
Papaveraceae	*Hypecoum procumbens* L. (LRSBG/AB/20/135)	جهيرة (الخشخاشية)	Aerial part	Can: 1* skin pimples and tumors	Raw	Topical
Parmeliaceae	*Evernia prunastri* L. (LRSBG/AB/20/158)	لحية شيخ	Lichens	Can: 1* cancer	Decoction	Oral
				GISD: 1* gastrointestinal diseases	Decoction	Oral
				NS: 1* epilepsy	Decoction	inhalation
Paronychioideae	*Telephium imperati* L. (LRSBG/AB/20/150)	تسمرغينت	Aerial part	GH: 1* mouth ulcer	Infusion	Topical/oral
				HSD: 1* anemia		
Pedaliaceae	*Sesamum indicum* L. (LRSBG/AB/20/136)	السِّمْسِم أو. جلجلان	Seeds	SRP: 2* infertility	Raw	Oral
				Breast milk outage	Maceration	Oral
				GH: 1* mouth ulcer	Decoction	Oral
Phyllanthaceae	*Phyllanthus niruri* L. (LRSBG/AB/20/137)	الأَمْلَج	Leaves	Can: 1* cancer	Raw/decoction	Oral
				RTD: 1* cough		
Pinaceae	*Pinus maritima* Aiton (LRSBG/AB/20/138)	الزنين	Fruits	Can: 2* blood cancer	Decoction	Oral
				Stomach cancer	Decoction	Oral
				Liver cancer	Decoction	Oral
				RTD: 1* chest and lung diseases	Maceration	Topical
				ESD: 1* goiter	Raw	Topical
				GH: 1* hoarseness and sore throat	Raw	Topical
	*Pinus pinaster* Aiton (LRSBG/AB/20/152)	تايدة لحاء شجرة الصنوبر البحري	Bark	GISD: 1* diarrhea	Raw	Oral
Piperaceae	*Piper cubeba* Bojer (LRSBG/AB/20/102)	الكبابة، حب العروس	Seeds	SRP: 1* infertility	Raw	Oral
	*Piper nigrum* L. (LRSBG/AB/20/103)	الفلفل الأسود	Seeds	GH: 1* earache and deafness	Decoction	Topical
Plantaginaceae	*Digitalis purpurea* L. (LRSBG/AB/20/115)	القِمَعية او الدِّيجيتال	Flowers	CVSD: 1* cardiovascular diseases	Raw	Oral
Poaceae	*Cymbopogon schoenanthus* (L.) Spreng. (LRSBG/AB/20/091)	الإذخر أو الليمونية	Leaves	Can: 1* skin pimples and tumors	Decoction	Topical
	*Stipa tenacissima* L. (LRSBG/AB/20/092)	نبات الحلفــــــــــــــاء	Leaves	CVSD: 1* cholesterol	Maceration	Oral
	*Hordeum vulgare* L. (LRSBG/AB/20/093)	الشعير الزرع	Seeds	SD: 1* burns	Frying	Topical
Poales	*Aristida pungens* Desf (LRSBG/AB/20/157)	الدرين	Stalk	HSD: 1* anemia	Decoction	Oral
Portulacaceae	*Portulaca oleracea* L. (LRSBG/AB/20/116)	البقلة او بندراق	Leaves	GISD: 1* stomach ache	Decoction	Oral
Punicaceae	*Punica granatum* L. (LRSBG/AB/20/080)	الرمان	Peels/fruits	GISD: 6* gastrointestinal diseases	Decoction	Oral
				IBS	Raw/decoction	Oral
				Heartburn	Stewing	Oral
				Stomach ache	Raw	Oral
				Diarrhea	Decoction	Oral
				GH: 2* mouth ulcer	Decoction	Topical
				Halitosis		
				NS: 1* headache	Decoction	Topical
Ranunculaceae	*Nigella sativa* L. (LRSBG/AB/20/139)	حبة البركة. أو الحبة السوداء. او السانوج	Seeds	NS: 1* migraine	Decoction	Inhalation
				RTD: 1* chest and lung diseases	Raw	Oral
				Can: 3* cancer	Raw	Oral
				SMSD: 3* acute arthritis and gout	Raw	Oral
				SD: 2* itchy skin	Raw	Oral
				Limb swelling		
Rhamnaceae	*Rhamnus alaternus* L*.* (LRSBG/AB/20/083)	آمليلس أو مليلس أو عود الخير	Bark/leaves/flowers	GISD: 2* jaundice and icterus	Decoction	Oral
	*Ziziphus spina-christi* (L.) Desf. (LRSBG/AB/20/084)	النبق شجرة السدر	Fruits/leaves	GISD: 1* jaundice and icterus	Raw	Oral
				Can: 1* cancer	Raw	Oral
				SD: 1* skin diseases and ulcer	Decoction	Oral
Rosaceae	*Potentilla reptans* L. (LRSBG/AB/20/166)	حشيشة الخامسة	Leaves	SD: 2* itchy skin limb swelling	Raw	Oral
					Raw	Oral
	*Prunus persica* (L.) Batsch. (LRSBG/AB/20/056)	الخوخ	Leaves	Can: 2* cancer	Raw	Oral
				SD: 1* limb swelling	Infusion	Topical
	*Alchemilla vulgaris* L. (LRSBG/AB/20/057)	رجل الأسد	Leaves	SD: 1* skin diseases and ulcer	Infusion	Topical
	*Crataegus azarolus* L. (LRSBG/AB/20/058)	الزعرور	Fruits/flowers	CVSD: 1* cardiovascular diseases	Raw	Oral
				NS: 2* headache	Decoction	Oral
				Dizziness		
	*Eriobotrya japonica* (Thunb.) Lindl	النيفلة او البشملة	Leaves	NS: 2* headache	Decoction	Oral
	(LRSBG/AB/20/059)			Dizziness		
	*Potentilla erecta* (L.) Raeusch. (LRSBG/AB/20/060)	لنجبار	Roots	SRP: 1* breast milk outage	Maceration	Oral
				RTD: 1* chest and lung diseases	Raw	Oral
				GISD: 2* stomach ache	Infusion	Oral
				Ulcers	Decoction	Oral
	*Prunus domestica* L. (LRSBG/AB/20/061)	البرقوق	Fruits	GISD: 2* jaundice	Maceration	Oral
				Liver diseases		
	*Prunus amygdalus* L. (LRSBG/AB/20/062)	اللوز	Fruits	SRP: 1* infertility	Decoction	Oral
	*Cydonia oblonga* Mill. (LRSBG/AB/20/063)	السَفَرْجَل	Fruits	CVSD: 1* cardiovascular diseases	Raw	Oral
Rubiaceae	*Rubia tinctorum* L*.* (LRSBG/AB/20/117)	الفُوَّة	Roots	NS: 1* sciatica	Raw	Oral
				SRP: 1* infertility	Raw	Oral
				HSD: 1* anemia	Maceration	Oral
				USD: 1* urinary tract infection/inflammation	Decoction	Oral
Rutaceae	*Citrus limon* (L.) Osbeck. (LRSBG/AB/20/105)	الليمون	Fruits	RTD: 3* asthma	Decoction	Oral
				Lung filtering/smoker	Decoction	Oral
				Pneumonia	Decoction	Oral
				NS: 1* dizziness	Decoction	Oral
				CVSD: 1* hypertension	Decoction	Oral
				GISD: 1* liver diseases	Decoction	Oral
				HSD: 1* spleen diseases	Decoction	Oral
	*Ruta chalepensis* L. (LRSBG/AB/20/106)	السَّذَاب أو الفَيْجَل	Aerial part	GISD: 2* IBS jaundice/icterus,	Decoction	Oral
					Decoction/raw	Oral/topical
				SRP: 6* infertility	Decoction	Topical
				GH: 1* earache and deafness	Infusion	Topical
				SD: 2* limb swelling	Raw	topical
				NS: 1* headache	Decoction	Oral
Salvadoraceae	*Salvadora persica* L. (LRSBG/AB/20/140)	مسواك	Bark	RTD: 2* asthma	Decoction	Oral
				Lung filtering/smoker		
				SRP: 1* infertility	Decoction	Oral
Santalaceae	*Santalum album* L. (LRSBG/AB/20/118)	الصَنْدَل	Bark/fruits	NS: 1* migraine	Decoction	Topical/oral
Scrophulariaceae	*Verbascum sinuatum* L. (LRSBG/AB/20/141)	مصلح الأنظار أو البوصير أو تيسراو	Leaves	RTD: 5* pneumonia, chest and lung diseases, and asthma	Infusion	Topical
				GISD: 6* IBS and stomach pain	Decoction	Oral and steam
Solanaceae	*Lycium shawii* Roem. and Schult (LRSBG/AB/20/142)	العوسج	Roots/fruits/leaves	SD: 5* skin ulcers	Decoction	Oral
				Leprosy		
				SRP: 4* uterine problems, infertility	Decoction	Oral
				SMSD: 2* osteoarthritis and gout	Decoction	Oral
	*Nicotiana tabacum* L. (LRSBG/AB/20/129)	الشمة	Leaves	GH: 1* tonsillitis	Infusion	Topical
Tamaricaceae	*Tamarix aphylla* (L.) H.Karst. (LRSBG/AB/20/143)	طحطاح	Leaves	NS: 1* headache	Decoction	Oral
Theaceae	*Camellia sinensis* (L.) Kuntze. (LRSBG/AB/20/120)	الشاي الأخضر	Leaves	SRP: 1* infertility	Maceration	Topical
				SD: 1* itchy skin	Maceration	Topical
Thymelaeaceae	*Daphne gnidium* L. (LRSBG/AB/20/107)	لازاز	Leaves	HC: 1* hair loss	Raw	Topical
				RTD: 1* sinusitis	Steaming	Topical
	*Aquilaria malaccensis* Lam. (LRSBG/AB/20/108)	العود الهندي أو عود غريس/أغريس	Bark	Can: 4* blood cancer	Decoction	Oral
				Stomach cancer	Decoction	Oral
				Liver cancer	Decoction	Oral
				Cancer	Raw	Topical
				HC: 1* alopecia areata	Raw	Oral
Ulmaceae	*Ulmus rubra* Muhl. (LRSBG/AB/20/144)	الدردار	Leaves	ID: 1* laryngitis	Decoction	Oral
				SMSD: 1* moving difficulty	Raw	Topical
Urticaceae	*Urtica dioica* L. (LRSBG/AB/20/121)	حُرَّيْق أو القُرَّاص	Leaves	USD: 1* urinary tract infection/inflammation	Decoction	Oral
				KD: 1* kidney problems	Decoction	Oral
				SMSD: 1* arthritis	Decoction	Oral
	*Parietaria officinalis* L. (LRSBG/AB/20/122)	فتات الحجر	Aerial part	KD: 1* urolithiasis	Decoction	Oral
Verbenaceae	*Verbena officinalis* L. (LRSBG/AB/20/146)	رِعْيُ الحَمَام	Aerial part	SRP: 1* uterine problems	Decoction	Oral
				USD: 1* bladder disease	Decoction	Oral
Vitaceae	*Vitis vinifera* L. (LRSBG/AB/20/147)	زبيب	Fruits	HSD: 1* anemia	Infusion	Oral
				SMSD: 2* back pain	Raw	Topical
				Moving difficulty	Raw	Oral
Xanthorrhoeaceae	*Aloe vera (L.)* Burm. f. (LRSBG/AB/20/164)	صبر	Leaves	SRP: 1* infertility	Decoction	Oral
				GISD: 1* stomach ache	Maceration	Oral
	*Aloe perryi* Baker (LRSBG/AB/20/125)	الصَبِر السُقُطْري	Leaves	GISD: 2* colitis + flatulence	Raw	Oral
Zingiberaceae	*Zingiber officinale Roscoe* (LRSBG/AB/20/064)	زنجبيل او سكنجبير	Roots	Can: 3* breast cancer	Raw	Topical
				Legs cancer	Maceration	Topical
				RTD: 2* chest and lung diseases	Decoction	Oral
				USD: 1* urinary tract infection/inflammation	Raw	Oral
				GISD: 3* colitis	Raw	Oral
				Flatulence	Raw	Topical
				Jaundice/icterus	Raw	Oral
				GH: 1* hoarseness and sore throat	Raw	Oral
				NS: 1* head problems and psychosis	Raw	Topical
				SD: 1* skin diseases and ulcer	Maceration	Topical
				ESD: 1* goiter	Decoction	Oral
	*Curcuma longa* L. (LRSBG/AB/20/065)	الكركم	Roots	GISD: 2* jaundice	Infusion	Oral
				Liver diseases	Infusion	Oral
				GH: 2* anxiety disorders and hypochondria	Raw	Topical/oral
				NS: 1* head problems and psychosis	Raw	Topical
	*Elettaria cardamomum* (L.) Maton. (LRSBG/AB/20/066)	حب الهال	Seeds	GISD: 1* heartburn	Decoction	Oral
Zygophyllaceae	*Tetraena alba* (L.f.) Beier and Thulin. (LRSBG/AB/20/148)	العكاية	Leaves/seeds	ESD: 1* diabetes	Decoction	Oral

Moreover, future ethnobotanical studies should adopt a multiple evidence-based approach that considers both the social-ecological-cultural context and local linguistic characteristics. In the same line, there is an urgent need for a clear strategy to include the local ethnobotanical knowledge in the conservation of biodiversity besides strong legislation aiming to protect the local medicinal species. Furthermore, establishing a unified local folk pharmacopeia based on different ethnobotanical and pharmacological investigations could be considered as one of the most important challenges in the future decade.

## Data Availability

The original contributions presented in the study are included in the article/Supplementary Material; further inquiries can be directed to the corresponding author.
